# Current status of electronic health literacy among pregnant women with gestational diabetes mellitus and their perceptions of online health information: a mixed-methods study

**DOI:** 10.1186/s12884-024-06594-w

**Published:** 2024-05-28

**Authors:** Jingqi Xu, Yujia Chen, Jing Zhao, Jiarun Wang, Jianfei Chen, Xinlong Pan, Wei Zhang, Jin Zheng, Zhijie Zou, Xiaoli Chen, Yingzi Zhang

**Affiliations:** 1 School of Nursing, Wuhan University, No. 115, Donghu Road, Wuhan, Hubei 430071 China; 2 Hospital of Stomatology, Wuhan University, 237 Luoyu Road, Wuhan, Hubei 430079 China; 3 Magnet Program & Nursing Research Department, UT Southwestern Medical Center, 8200 Brookriver Dr, Dallas, TX 75247 USA

**Keywords:** Gestational diabetes mellitus, Electronic health literacy, Online health information, Pregnant women

## Abstract

**Background:**

Women diagnosed with gestational diabetes mellitus often rely on internet-based health information for managing their condition. This study aims to investigate the present state of electronic health literacy among women with gestational diabetes mellitus, analyze the influencing factors, and explore their experiences regarding accessing, comprehending, evaluating, and applying online health information pertinent to gestational diabetes mellitus.

**Methods:**

A sequential explanatory mixed methods research design was adopted in this study. Initially, 235 women with gestational diabetes mellitus participated in a cross-sectional survey. The research tools included general information and the Chinese version of the electronic Health Literacy Scale (eHEALS). Descriptive analyses were conducted to describe the characteristics of the sample, and multiple linear regression analyses were used to explore the factors influencing electronic health literacy among women with gestational diabetes mellitus. Secondly, 11 women with gestational diabetes mellitus joined semi-structured in-depth interviews to obtain their perceptions about online health information. The data were analyzed using inductive content analysis to develop themes.

**Results:**

The median score of eHEALS in the Chinese version among 235 women diagnosed with gestational diabetes mellitus was 29 (interquartile range [IQR], 26 to 32). Factors influencing electronic health literacy among these women included accessing health information from medical professionals (β = 0.137, *p* = 0.029) and utilizing health information from applications (β = 0.159, *p* = 0.013). From the qualitative phase of the study, four thematic categories emerged: reasons and basis for accessing health information from the Internet; address barriers to accessing and applying online health information; desires for a higher level of online health information services; outcomes of accessing and applying online health information.

**Conclusion:**

The electronic health literacy of women diagnosed with gestational diabetes mellitus remains suboptimal and warrants improvement. The sources of access to health information affect electronic health literacy in women with gestational diabetes mellitus. Moreover, women facing gestational diabetes encounter numerous impediments when attempting to access health-related information online, underscoring the necessity for enhanced online health information services to meet their needs.

**Supplementary Information:**

The online version contains supplementary material available at 10.1186/s12884-024-06594-w.

## Background

Gestational diabetes mellitus is a metabolic disorder occurring during pregnancy [1], primarily resulting from insulin resistance and the progressive dysfunction of pancreatic β-cell [2]. Symptoms of gestational diabetes mellitus often manifest insidiously, making detection challenging. Diagnosis typically occurs through the oral glucose tolerance test administered between the 24th and 28th weeks of gestation [3]. Although there have been some advancements in monitoring the fetal health of women with gestational diabetes [4, 5], gestational diabetes mellitus remains one of the most important causes of adverse perinatal outcomes [6, 7], which may also have a negative impact on maternal mental health [8]. To mitigate these adverse effects, a collaborative multidisciplinary approach is typically employed, with lifestyle and behavioral management serving as the preferred method of intervention [9]. Lifestyle and behavioral management strategies for gestational diabetes mellitus encompass a diverse array of medical knowledge, spanning medical nutrition therapy, physical activity recommendations, weight management strategies, and more [10]. Therefore, to effectively manage gestational diabetes mellitus, women typically require access to extensive health information regarding lifestyle and behavioral management strategies.

In recent years, with the development of information and communication technologies, electronic resources have been increasingly used in healthcare. The Internet, in particular, has emerged as a popular platform for accessing health information among women diagnosed with gestational diabetes mellitus [11]. However, despite the convenience afforded by the Internet for accessing health information, it is essential to acknowledge the challenges associated with online health information and services. These challenges include content duplication, the presence of unregulated information sources, inadequate quality control measures, and difficulty in verifying the credibility of information sources [12]. Therefore, for women managing gestational diabetes mellitus, discerning the most reliable and credible health information from the vast array of online resources is paramount.

According to Norman and Skinner, the ability of individuals to access reliable and credible health information from electronic resources hinges on their electronic health literacy, an extension of traditional health literacy within the digital realm [13]. Unlike traditional health literacy, which primarily emphasizes individual access to and understanding of health information [14], electronic health literacy focuses on the individual comprehensive ability to access, understand, and assess health information from electronic resources, and apply health information available online to address health issues or make health-related decisions [15]. Evidence suggests that individual electronic health literacy is positively associated with one’s health behaviors and health outcomes, including a higher level of medication adherence, psychosocial well-being, and quality of life, as well as adopting adaptive health behaviors [16–19]. Therefore, to enhance the health behaviors and outcomes of women diagnosed with gestational diabetes mellitus, a thorough understanding of their electronic health literacy is indispensable.

Most of the existing studies on electronic health literacy focus on adolescents, college students, and the elderly [20–22]. In recent years, a few researchers have explored electronic health literacy in people with chronic diseases and their caregivers, including cancer patients and their caregivers [23, 24], individuals with systemic lupus erythematosus, and those diagnosed with diabetes [25]. To the best of our knowledge, there is relatively limited research on the electronic health literacy of pregnant women, and currently, no studies have investigated the electronic health literacy of women with gestational diabetes mellitus. Through a review of studies on electronic health literacy in other populations, it was found that demographic characteristics, pregnancy-related features, and sources of health information acquisition may influence the electronic health literacy of women with gestational diabetes mellitus, including factors such as age, education level, employment status, household income, residential location, gestational age, number of pregnancies, and online health information searching [26–31]. In addition, research on electronic health literacy is primarily quantitative, while comprehensive studies on the experience and needs related to electronic health information remain insufficient. Taking these factors into consideration, this study adopted a mixed-methods approach to investigate electronic health literacy among women with gestational diabetes mellitus. It thoroughly explored the factors that influence electronic health literacy in this population, while also delving into their experiences of accessing, comprehending, evaluating, and applying online health information. Based on the literature review above, before the study began, we hypothesized that demographic characteristics, pregnancy-related factors, and sources of health information acquisition are associated with the electronic health literacy of pregnant women with gestational diabetes.

## Method

### Design

A sequential explanatory mixed-methods research design was employed to investigate the current status of electronic health literacy and cognition of online health information among women diagnosed with gestational diabetes mellitus. This study is divided into two parts. The first part discusses the current status and influencing factors of electronic health literacy among women with gestational diabetes mellitus through quantitative analysis. In the second part, qualitative research was conducted to explore the perception and cognition of women with gestational diabetes mellitus on online health information.

### Quantitative phase—questionnaire survey

#### Study design and setting

The quantitative phase is a cross-sectional study conducted through questionnaire surveys. During this phase, we recruited pregnant women diagnosed with gestational diabetes mellitus from the obstetrics department of a tertiary maternity hospital in Wuhan City using a convenience sampling method. The inclusion criteria were as follows: (1) aged 18 years old and above; (2) diagnosed with gestational diabetes mellitus according to the International Association of Diabetes and Pregnancy Study Groups (IADPSG) criteria; (3) native Chinese speakers or non-native Chinese speakers who could understand Chinese well; (4) no cognitive impairment and normal mental state; (5) signed informed consent. Exclusion criteria included the inability to complete the questionnaire due to poor physical condition.

#### Sample

The sample size for studies on variable influencing factors should be determined according to the requirements of statistical variable analysis, typically recommended to be at least 5 to 10 times the number of variables [32]. In this study, based on 19 variables (16 independent variables and the 3 dimensions of the electronic health literacy scale), the estimated sample size ranged from 95 to 190. Considering a 20% invalid questionnaire rate, this section ultimately included 235 participants.

#### Data collection

Data were obtained through a self-completed questionnaire between July 20, 2022 and September 10, 2022. The questionnaire included the collection of independent and dependent variable information. The collection of independent variable information was based on a review of previous studies, covering general data related to demographic characteristics, pregnancy features, and sources of obtaining healthcare information. The instrument for collecting dependent variable information is the Chinese version of the eHEALS.

The eHEALS is the original and most frequently used instrument for investigating electronic health literacy [33]. It was initially developed by Norman and Skinner in 2006 [34]. The Cronbach alpha coefficient of the original English version of eHEALS is 0.88. The Chinese version of eHEALS was translated by Guo in 2013 [35]. It consists of 3 dimensions with 8 items, scored on a 5-point Likert scale. The score of each item ranges from 1 (strongly disagree) to 5 (strongly agree), with higher scores indicating greater electronic health literacy. The Chinese version of eHEALS demonstrates good reliability and validity. Regarding reliability, the Cronbach’s α coefficient is 0.913 [35]. For validity, exploratory factor analysis reveals a KMO coefficient of 0.875 and a significant Bartlett’s test of sphericity with a χ2 value of 544.000 (df = 28); confirmatory factor analysis indicates factor loadings ranging from 0.692 to 0.869 [35]. In our study, the Cronbach’s alpha coefficient for eHEALS was 0.937.

#### Data analysis

IBM SPSS Statistics was employed for statistical analysis. Demographic and pregnancy characteristics of participants were presented using descriptive statistics. Continuous variables were described by means and standard deviations, or medians and interquartile, depending on the normality of the data. Categorical variables were described by frequencies and percentages. To investigate the correlation between general data and e-health literacy among pregnant women, univariate analysis was performed. Due to the non-normal distribution of the data, either the Mann-Whitney U test or Kruskal-Wallis H test was utilized. Subsequently, the general data of women with gestational diabetes mellitus (*p* < 0.05) from the univariate analysis were included as independent variables in a multiple linear regression model, with e-health literacy as the dependent variables, to explore the influencing factors of e-health literacy.

### Qualitative phase—in-depth interviews

#### Study design and sample

Qualitative data was collected through semi-structured in-depth interviews between September 1, 2022, and October 3, 2022. The sample size was determined based on the saturation principle, which means that sample recruitment continued until no new codes emerged [36]. Ultimately, a total of 11 participants were enrolled. Among these, four participants took part in both the qualitative and quantitative segments of the study, while the remaining seven exclusively contributed to the qualitative phase.

#### Data collection

Before the interviews began, a survey was conducted on the personal basic information and electronic health literacy status of all 11 participants involved in the interviews.

The semi-structured interview instrument comprised 10 questions (Supplementary 1). The interview location was a quiet and clean reception room for pregnant women at the obstetrics clinic, which ensured the privacy of the interviews. Two researchers were involved: one recorded environmental information, interviewees’ non-verbal communication, and facial expressions, while the other conducted the interviews with pregnant women. Midway through the study, owing to the COVID-19 pandemic, researchers conducted interviews with pregnant women via online video calls. All interviews were audio-recorded and transcribed verbatim.

#### Data analysis

The qualitative data from 11 interview transcripts were coded using NVivo 11.0, and analyzed using the inductive content analysis method described by Elo and Kyngäs [37]. The process of inductive content analysis comprises three phases. Open coding (Phases 1): researchers immersed themselves in the text data, generating numerous notes and headings to capture the content comprehensively. Subsequently, the researchers organized the headings into coding sheets and freely generated categories. Creating categories (Phases 2): the researchers amalgamated akin or disparate categories into higher-order categories for reducing the number of categories. Abstraction (Phases 3): the researchers delineated research topics through the utilization of generalized descriptions, thereby shaping the themes.

## Results

### Quantitative results

#### Description of the sample

The eHEALS score in the Chinese version, obtained from 235 women diagnosed with gestational diabetes mellitus, spanned from 8 to 40, with a median score of 29 (IQR, 26 to 32). The median age of these participants was 31 (IQR, 29 to 34) years and their median gestational age was 34 (IQR, 32 to 36) weeks. All individuals involved in the study identified as Han Chinese. Further demographic and pregnancy characteristics of participants are shown in Table 1.

**Table 1:** Characteristics of participants and the eHEALS scores for participants with different characteristics (*n* = 235)

Characteristics	Categories	*n* (%)	Score of eHEALS, M (ICQ)	Z/H	*P*
**Demographic characteristics**					
Age	< 35	186 (79. 1%)	29 (26–32)	−0.758	0.448^a^
	≥ 35	49 (20.9%)	30 (26–34)		
Educational status	Junior high school and below	34 (14.5%)	27 (25–30)	14.001	0.003^b^
	High school or technical secondary school	18 (7.7%)	26 (24–33)		
	Junior college or undergraduate	155 (66.0%)	29 (26–32)		
	Master degree and above	28 (11.9%)	33 (30–37)		
Long-term residence	Cities and towns	208 (88.5%)	30 (26–33)	− 1.301	0.193^a^
	Rural	27 (11.5%)	27 (24–32)		
Average income per person in family, RMB	< 5000	36 (15.3%)	29 (26–32)	5.589	0.133^b^
	5000–7499	62 (26.4%)	29 (26–32)		
	7500–9999	64 (27.2%)	29 (24–32)		
	≥ 10,000	73 (31. 1%)	31 (26–36)		
Family history of diabetes	Yes	84 (35.7%)	29 (26–32)	−0.684	0.494^a^
	No	151 (64.3%)	30 (25–34)		
Type I diabetes, type 2 diabetes, polycystic ovary syndrome	Yes	28 (11.9%)	31 (29–33)	− 1.310	0.190^a^
	No	207 (88. 1%)	29 (25–32)		
**Pregnancy characteristics**					
Gravidity, n (%)	First	124 (52.8%)	31 (26–33)	− 1.440	0.150^a^
	≥ 2	111 (47.2%)	29 (25–32)		
Parity, n (%)	Nulliparous	133 (56.6%)	30 (26–33)	− 1.033	0.302^a^
	≥ 1	102 (43.4%)	29 (26–32)		
Adverse outcomes in previous pregnancies ^c^	Yes	56 (23.8%)	29 (24–32)	− 1.886	0.059^a^
	No	179 (76.2%)	30 (26–33)		
Existing pregnancy complications or comorbidities ^d^	Yes	73 (31.1%)	29 (25–34)	−0.341	0.733^a^
	No	162 (68.9%)	30 (26–32)		
**Sources of access to health information**					
Clinicians or nurses	Yes	213 (90.6%)	30 (26–33)	−2.289	0.022^a^
	No	22 (9.4%)	28 (24–30)		
Books or newspapers	Yes	27 (11.5%)	31 (26–35)	−0.878	0.380^a^
	No	208 (88.5%)	29 (25–32)		
Social forums or WeChat official accounts	Yes	64 (27.2%)	31 (27–35)	−2.374	0.018^a^
	No	171 (72.8%)	29 (25–32)		
Applications	Yes	148 (63.0% )	30 (26–34)	−2.398	0.016^a^
	No	87 (37.0%)	29 (25–32)		
Internet pages	Yes	88 (37.4%)	30 (27–34)	− 1.998	0.046^a^
	No	147 (62.6%)	29 (25–32)		
Satisfaction with health information on the Internet	Extremely dissatisfied	4 (1.7%)	29 (13–33)	15.227	0.002^b^
	Slightly dissatisfied	19 (8. 1%)	25 (23–28)		
	Slightly Satisfied	187 (79.6%)	30 (26–32)		
	Very satisfied	25 (10.6%)	32 (25–40)		

M (IQR) means median (interquartile range)

a The Mann-Whitney test

b The Kruskal-Wallis test

c Adverse outcomes in previous pregnancy includes extrauterine pregnancy, premature labour spontaneous abortion, stillbirth, macrosomia, hypothyroidism, hyperthyroidism, GDM, gestational hypertension, etc.

d Existing pregnancy complications or comorbidities includes gestational hypertension, pre-eclampsia, threatened miscarriage, severe anemia, hypothyroidism, hyperthyroidism, intrahepatic cholestasis of pregnancy, etc.

#### Influencing factors of electronic health literacy in women with gestational diabetes mellitus

The results of single factor analysis indicated that educational status (*p* = 0.003), experience of accessing health information from clinicians or nurses (*p* = 0.022), experience of accessing health information from social forums or WeChat official accounts (*p* = 0.018), experience of accessing health information from applications (*p* = 0.016), experience of accessing health information from Internet pages (*p* = 0.046), and satisfaction with health information on the Internet (*p* = 0.002) had a statistically significant difference in electronic health literacy scores of women with gestational diabetes mellitus. The results are shown in Table 1. Additionally, correlation analysis of gestational weeks and electronic health literacy scores showed that gestational weeks and electronic health literacy were not correlated in women with gestational diabetes mellitus (*p* = 0.346).

In the multiple linear regression analysis, the eHEALS score served as the dependent variable, while the statistically significant factors identified in the univariate analysis were considered independent variables. *P* < 0.05 indicates statistical significance. Results showed that women with gestational diabetes mellitus who accessed health information from clinicians or nurses scored higher on the eHEALS than those who did not (β = 0.137, *p* = 0.029). Similarly, women with gestational diabetes mellitus who accessed health information from applications demonstrated higher eHEALS scores than those who did not do (β = 0.159, *p* = 0.013). These results are shown in Table 2.

**Table 2:** Multiple linear regression analysis of eHealth literacy in women with GDM

Variables	B	SE	β	t	*P*
(Constant) Educational status	19.382	3.275		5.917	< 0.001
Junior high school and below	Reference				
High school or technical secondary school	−0.394	1.707	−0.017	−0.231	0.818
Junior college or undergraduate	0.493	1.145	0.038	0.431	0.667
Master degree and above	3.050	1.560	0.161	1.955	0.052
Sources of access to health information					
Clinicians or nurses					
No	Reference				
Yes	2.884	1.310	0.137	2.202	0.029
Social forums or WeChat official accounts					
No	Reference				
Yes	0.994	0.904	0.072	1.100	0.272
Applications					
No	Reference				
Yes	2.013	0.807	0.159	2.494	0.013
Internet pages					
No	Reference				
Yes	1.338	0.804	0.106	1.665	0.097
Satisfaction with health information on the Internet					
Extremely dissatisfied	Reference				
Slightly dissatisfied	5.860	3.168	0.295	1.850	0.066
Slightly satisfied	4.831	2.982	0.318	1.620	0.107
Very satisfied	0.894	3.235	0.040	0.276	0.783

*Note B*, unstandardized coefficient of regression; *β*, standardized coefficient of regression, *R* = 0.391, R2 = 0.153, adjusted R2 = 0.115, F = 4.042, *p* < 0.001

### Qualitative findings

A total of 11 women with gestational diabetes mellitus participated in the interviews, designated with identifiers P1 to P11 based on the interview sequence. All interviewees were married and of Han nationality. Their age ranged from 27 to 36 years, with an average age of approximately 31 years. Three participants were in their second trimester, while the remaining were in their third trimester. Notably, only one interviewee, identified as P1, had prior pregnancy experience and already had one child. Furthermore, the ninth participant possessed a medical background and resided in a rural area. Among the participants, five individuals scored 32 points or more on the Chinese version of eHEALS (The score of eHEALS range from 26 to 40). The general information about the participants is presented in Supplementary 2.

Based on the results of the interviews, a total of 4 themes and 12 sub-themes were identified. Supplementary 3 presents excerpts of selected quotes corresponding to each theme.

#### Reasons and basis for accessing health information from the internet

This theme revealed why and how women with gestational diabetes mellitus access health information from the Internet. They access information pertaining to maintaining a healthy pregnancy, managing their condition, monitoring fetal growth and development, and ensuring a successful delivery by utilizing Internet searches or subscribing to popular medical science articles disseminated via WeChat official accounts and pregnancy-related applications. The preference for electronic media among women with gestational diabetes mellitus is influenced by factors such as their previous information-seeking habits, recommendations from friends, and insights derived from data analysis. These information-seeking behaviors are motivated by concerns regarding health risks associated with disease exposure and perceived barriers to effective doctor-patient communication.

##### Reasons for accessing health information from the internet

The majority of interviewees reported actively seeking or passively receiving health information from the Internet. Their motivations included encountering abnormal prenatal examination results, experiencing personal or family physical discomfort, and lacking sufficient knowledge about various medical conditions.

Furthermore, some interviewees highlighted communication barriers between healthcare providers and patients, including distrust of doctors, dissatisfaction with their performance, and the impact of the COVID-19 pandemic, as factors prompting them to resort to the Internet for health information.

##### Basis for selecting electronic media providing health information

The interviewees utilize diverse electronic media platforms like Baidu, Little Red Book, and Baby Tree for accessing health information. Their choices are frequently influenced by previous preferences, recommendations from acquaintances, and the promotion of big data.

#### Address barriers to accessing and applying online health information

Many barriers impede women with gestational diabetes mellitus in accessing and applying health information available online, including advertising, inappropriate medical depth of health information, redundant and cluttered health information, conflicting opinions on the same health issue, wide period and content span for health information update, and difficulties in evaluating the quality, sources, and safety of online health information. In response, they adopted strategies to address these barriers, including asking for help, exploring and practicing independently, and assessing the credentials of health information providers.

##### Barriers abound

During the interviews, women with gestational diabetes mellitus indicated that they encountered many barriers in accessing information. Two interviewees noted excessive hidden advertisements in online health information. Additionally, two interviewees pointed out that the medical depth of the health information available online was inappropriate and they expressed that this health information was insufficient to address their health concerns. Furthermore, three interviewees expressed difficulty in making decisions due to the plethora of conflicting opinions encountered online regarding the same health issue. Two respondents highlighted that the frequency and scope of updates to online health information posed obstacles to their access. Three respondents expressed apprehensions regarding the quality, source, and safety of the information available online.

##### Respond to barriers

Whenever women with gestational diabetes mellitus encounter difficulties accessing valuable health information online or have doubts about the reliability of the information they find, they tend to seek guidance from individuals with more expertise or experience, such as hospital doctors, online healthcare professionals, and peers who have similar experiences. They said that if they did not know whether health information available online was credible, they would try to practice it personally and judge the truth of health information based on their health changes. In addition, they expressed that they would try to retrieve health information through multiple online sources, compare the information content, and finally trust the highly overlapping parts. Furthermore, they also evaluate the credibility of online health information by assessing the credentials of information providers.

#### Desires for a higher level of online health information services

Women diagnosed with gestational diabetes mellitus often turn to the Internet as a supplementary resource for obtaining health-related information, yet deficiencies persist within current online health information platforms. Their expressed aspirations for enhanced online health services manifest across four key dimensions, as outlined below.

##### Desires for online transmission media with simple design and easy-to-use search function

Women diagnosed with gestational diabetes mellitus express a preference for online health information platforms that prioritize user-friendly design and enhanced searchability. Such features streamline software navigation, thereby facilitating their information retrieval process.

##### Desires for diversified online transmission forms of health information

Women diagnosed with gestational diabetes mellitus expressed a clear preference for online health information dissemination to encompass not only simple textual descriptions but also incorporate videos and images, thereby enhancing the comprehensibility and appeal of the content.

##### Desires for online information platforms containing real cases and experience sharing

Women diagnosed with gestational diabetes mellitus articulated the wish for web-based platforms to feature shared experiences from pregnant women and real-life cases. This inclusion is seen as instrumental in fostering confidence in recovery, accessing credible health information, and gaining deeper insights into pregnancy-related matters.

##### Desires for online information platforms with strong interactivity and personalized health information push services

Women with gestational diabetes mellitus expressed their desire for the personalized push service of health information provided by the web-based platforms, preferably sending health information according to their pregnancy duration. They also seek increased interaction with medical professionals on web-based platforms to receive more personalized and relevant advice and guidance.

#### Outcomes of accessing and applying online health information

Women with gestational diabetes mellitus noted that applying and accessing online health information could not only enhance their health literacy but also foster greater awareness of adopting a healthy lifestyle and encourage increased involvement from their spouses. However, they also acknowledged potential adverse effects, such as heightened anxiety stemming from the treatment experiences shared by others.

##### Popularization of health knowledge

Women with gestational diabetes mellitus point out that accessing online health information has improved their health knowledge and helps them effectively control blood sugar levels.

##### Emotional feedback

Some women diagnosed with gestational diabetes mellitus remarked that the severity of the condition was often exaggerated on the Internet, leading to heightened anxiety. Furthermore, encountering accounts of successful disease management shared by others sometimes evoked feelings of self-doubt regarding their own ability to manage the condition, consequently causing stress and anxiety. Conversely, one woman with gestational diabetes mellitus expressed that upon encountering individuals facing similar health challenges online, she found solace in the shared experience of others facing similar struggles.

##### Increased awareness about adapting healthy lifestyles

Women diagnosed with gestational diabetes mellitus emphasized that their awareness of adopting healthy lifestyles had been heightened through their exploration of health information accessible on the Internet.

##### Increased husband’s sense of involvement and experience

Women diagnosed with gestational diabetes mellitus noted that their husbands also have the opportunity to access online health information, thereby enabling them to gain a deeper understanding of the pregnancy experience.

## Discussion

To the best of our knowledge, this is the first study to investigate electronic health literacy among women with gestational diabetes mellitus through a mixed-methods design. Our study indicates that the electronic health literacy of women with gestational diabetes warrants improvement. Additionally, we delved into reasons for seeking health information online, barriers encountered, aspirations for improved online health services, and the impacts of utilizing online health information.

In terms of the influencing factors on electronic health literacy, our results indicated that women with gestational diabetes mellitus who accessed health information from medical personnel scored higher on electronic health literacy compared to those who did not, which was inconsistent with Kim et al.‘s finding that there was no difference in electronic health literacy scores between those with type 2 diabetes who relied on health professionals for health information and those who did not [38]. One possible explanation for this discrepancy is the variation in disease self-management capabilities. The majority of people with type 2 diabetes surveyed had managed their diabetes for 1–10 years, while participants in our study were diagnosed with gestational diabetes for a maximum of three months. The duration of illness positively correlates with the level of self-management [39]. This suggests that gestational diabetes patients may have weaker disease self-management abilities compared to type 2 diabetes patients, leading to a greater need for healthcare professionals’ assistance in addressing more health issues and facilitating gestational diabetes women’s understanding and application of online health information [40]. Additionally, the reason for this outcome in our study may be attributed to inadequate communication between healthcare professionals and patients [41]. Evidence suggests that individuals turn to the internet for information when their health concerns are not addressed by healthcare providers during consultations [41]. In the qualitative portion of our study, some patients reported that their issues were not fully resolved after communication with healthcare providers or that new uncertainties arose from these interactions. Consequently, women diagnosed with gestational diabetes mellitus turn to the internet as an additional resource for health information, thereby augmenting their level of electronic health literacy [42].

The control of blood sugar levels is crucial for women with gestational diabetes mellitus, and continuous blood sugar monitoring, along with maintaining a healthy diet and lifestyle, is key to controlling blood sugar [43–46]. Our research findings indicate that by accessing online health information, women with gestational diabetes mellitus can gain a deeper understanding of information related to blood sugar control, thereby effectively managing their blood sugar levels. Amr Jamal et al. have also noted that patients who engage in online health information queries have a better understanding of diabetes-related knowledge and demonstrate stronger blood sugar management capabilities compared to those who do not [47]. Therefore, future research should continue to explore the impact of this online health information on blood sugar management among women with gestational diabetes mellitus, thus effectively improving the management and prognosis of the disease.

Studies have demonstrated that precise health guidance aids in both treating gestational diabetes and preventing its development in high-risk pregnant women [48, 49]. Although the qualitative results of this study indicate that online health information searches play a role in health guidance, this depends on the quality of the information obtained. Accurate online medical information can assist patients in comprehending their condition and guide them toward suitable treatment options [50]. However, inaccurate or misleading information can result in confusion and treatment delays [51]. The results of our qualitative study showed that women with gestational diabetes mellitus were not competent in discerning the quality of health information available online. Therefore, it is necessary to evaluate the quality of online health information. Presently, several tools have been developed to assess the quality of websites providing health information, including DISCERN, HONcode, and CRAAP [52]. However, current investigations into the quality of online health information primarily focus on cancer patients [53–55], with relatively limited research on the quality of online health information for gestational diabetes. Future studies could address this gap to assist gestational diabetes women in better selecting online health information. Additionally, the authority of online health information publishers has a positive impact on the credibility of health information [56]. Medical professionals have traditionally been the primary source of health information for individuals, being widely regarded as the most authoritative [57]. In our study, participants expressed a greater willingness to trust online health information published by certified healthcare professionals. These indications suggest the necessity of encouraging healthcare professionals to take responsibility for providing online guidance and support to women with gestational diabetes, thereby facilitating their access to and utilization of high-quality online healthcare information.

In terms of the design of online health platforms, interviewees expressed desires for easy access to health information, receiving personalized push services of health information, and increased interaction with medical personnel through these platforms, aligning with findings by Nijland et al. [58]. These implied that at the outset of developing online health information platforms, platform designers need to consider how to deliver health information to users in an understandable and accessible manner, as well as how to tailor health information to users’ needs [59].

Due to the impact of the COVID-19 pandemic, we chose to conduct online video interviews with some participants. Compared to traditional offline interviews, online interviews offer more convenience in terms of time and space, but they also present some challenges [60]. Firstly, there are issues with internet connectivity, as online video interviews may be affected by network interruptions, thus disrupting the smooth progress of the interviews [61]. Secondly, online video interviews lack the emotional connection and interpersonal interaction of face-to-face communication, which may affect the richness of the information provided by the interviewees [62]. Lastly, due to issues with image quality and angles, online video interviews may not accurately capture the facial expressions and body language of the interviewees, thereby impacting the understanding and interpretation of the interview information [63]. The epidemic has sparked increased interest in video interviews, but video interviews should not be seen solely as expedient measures in response to the pandemic, but rather as an opportunity for long-term methodological advancement. Future research should further optimize the process of online video interviews to facilitate the development of virtual qualitative research methods.

## Limitations

Some limitations needed to be reported. Firstly, the quantitative study utilized a self-assessment scale as the research instrument. Participants may have either exaggerated or minimized certain information to obtain more favorable results, potentially introducing reporting bias. Secondly, all participants were sourced from a single hospital, potentially impacting the generalizability of the findings. Lastly, participants who engaged in both quantitative and qualitative phases of the study appeared more prepared at qualitative interviews compared to those solely involved in the qualitative phase. This discrepancy may introduce bias into their responses.

## Conclusions

Women with gestational diabetes mellitus have a low level of electronic health literacy and insufficient ability to assess online health information, and the source of health information could influence their electronic health literacy. They often accessed health information from the Internet due to perceived disease threats and blocked doctor-patient communication. Furthermore, they highlighted numerous barriers to accessing electronic health information and expressed a desire for enhanced quality in online information services. It is recommended to enhance doctor-patient communication and encourage medical staff to take on a guiding and supportive role to facilitate access to valuable information. Additionally, the development of assessment tools tailored to online health information suitable for women with gestational diabetes mellitus is proposed. Furthermore, improvements to online health information platforms are suggested to better align with user needs, thereby enhancing the electronic health literacy of women diagnosed with gestational diabetes mellitus.

### Electronic supplementary material

Below is the link to the electronic supplementary material.

Supplementary Material 1

## Data Availability

Owing to the confidentiality of the information, the datasets generated and analyzed in this study are not publicly available. Nevertheless, upon reasonable request, they can be made accessible through the corresponding author.
