# Supplementary material for: Current status of electronic health literacy among pregnant women with gestational diabetes mellitus and their perceptions of online health information: a mixed-methods study
Source: BMC Pregnancy Childbirth. 2024 May 28;24:392. doi: 10.1186/s12884-024-06594-w (PMC11134622; doi:10.1186/s12884-024-06594-w)
Supplement: Supplementary file 1 — Supplementary Material 1 [file 12884_2024_6594_MOESM1_ESM.docx]

**Supplementary 1:**

**Interview outline:**

1. In what situations do you go online to find health information?

2. How do you find health information online?

3. How do you interpret the health information you get from the internet?

4. What do you think of the quality of health information available online? Why?

5. How do you assess the quality of health information you get from the Internet?

6. How do you apply the health information you get online to your life?

7. What do you think are the factors that prevent you from accessing, understanding, and evaluating online health information?

8. What do you think are the factors that motivate you to access, understand, and evaluate online health information?

9. Have you experienced any difficulties in applying health information obtained from the Internet?

10. In what areas do you think the existing online health information platform needs to be improved?

| **Supplementary 2** General information of participants | | | | | | | | |
| --- | --- | --- | --- | --- | --- | --- | --- | --- |
| No | Age | Gestational  weeks | Educational  status | Number of  children  already | Gravidity | Average income per person in family (RMB) | Existing  pregnancy  Complications or comorbidities | Scores of eHEALS |
| P1 | 36 | 30 | Junior college or undergraduate | 1 | Second | 5000-7499 | None | 33 |
| P2 | 27 | 34 | Junior college or undergraduate | 0 | first | 7500-9999 | None | 36 |
| P3 | 29 | 26 | Junior college or undergraduate | 0 | first | ≥10000 | None | 31 |
| P4 | 28 | 35 | Junior college or undergraduate | 0 | first | 5000-7499 | Hypothyroidism | 40 |
| P5 | 34 | 32 | Junior college or undergraduate | 0 | first | 5000-7499 | None | 36 |
| P6 | 31 | 38 | Junior college or undergraduate | 0 | first | 7500-9999 | None | 32 |
| P7 | 30 | 34 | Master degree and above | 0 | first | ≥10000 | None | 28 |
| P8 | 30 | 34 | Junior college or undergraduate | 0 | first | 5000-7499 | None | 30 |
| P9 | 33 | 34 | Junior college or undergraduate | 0 | first | <5000 | None | 29 |
| P10 | 32 | 33 | Junior college or undergraduate | 0 | first | 7500-9999 | Hypothyroidism | 26 |
| P11 | 27 | 34 | Junior college or undergraduate | 0 | first | 7500-9999 | None | 29 |

| **Supplementary 3** Qualitative themes and quotes. | |
| --- | --- |
| Categories | quotes |
| 1. Reasons and basis for accessing health information from the Internet |  |
| 1. Reasons for accessing health information from the Internet | *I search on Zhihu, Bilibili, or Baidu. Then, what was on the wechat public account, what did I read. Here is the thing. Another is that if there is any diseases in my families, I would pay more attention to these information and then search. (P8)*  *I only paid attention to these things after I was pregnant. I didn't pay much attention to these (health information) because I was in good health before. After pregnancy, it was mainly because my glucose was a bit high. I failed my glucose tolerance test. Two-hour postprandial blood glucose did not meet the standard, so I was more concerned about how to control my glucose. I read a lot things on the Internet. (P11)*  *After I went to the hospital, the doctor told me that there was something wrong with me. But, I didn't know much about the disease, so that I would like to learn more about the disease through some app or through something on the Internet. (P2)*  *I feel that they are not willing to talk much, so I may spend more time to searching on my own. I didn't feel like talking to doctors too much as long as there was nothing too serious in the examination report. They just prescribe inspection and medicine. This is my true idea. (P6)*  *Sometimes they speak very fast, and then they are in a hurry when the next person arrives. Sometimes there may be some such situations. But, we don 't always understand and remember... Talking extra fast! You might as well make an applet of WeChat directly. Because you look at things like these on the Internet, such as the issue of blood glucose, what to eat and what to drink, and then how much blood glucose should be, it tells everyone clearly. (P11)*  *Because during the epidemic now, the maternity check-ups won't be as normal as usual. I won't be going to the hospital as often and it's not easy for me to go out, so I will read some knowledge written in Baby Tree and Mum Tree. (P11)* |
| 1. Basis for selecting electronic media providing health information | *I more usually use Baidu, because we usually search what we don’t know through Baidu. Then, because Douyin is now a relatively commonly used application. There is a lot of information available in Douyin, so this platform was used. (P10)*  *This is what I have heard from others. Classmates and friends who have been pregnant, they recommended it. (P4)*  *I actually found these two apps unintentionally. In fact, there may be an aspect that their advertising is a little more attractive, and then in the place of downloading software, they ranked a little bit in front, and then I also casually downloaded. (P1)* |
| 1. Address barriers to accessing and applying online health information |  |
| 1. Barriers abound | *Whether in Little Red Booklittle, DouYin or Baidu, there are some other miscellaneous advertisements....You have read very seriously, but found that it is to push a product for you. (P4)*  *I don't trust Baidu very much anyway, and there are too many advertisements. Sometimes you enter advertisement interface without noticing. (P11)*  *I can understand information on the Internet as long as it is not particularly professional.* *Like the kind of information that doesn't require a medical background to understand, I can basically understand it. (P7)*  *I feel like there are some questions on the Internet were asked simply, and then the doctors answered them simply, not in too much detail. The doctors’ answer is very broad, for example, blood sugar. They may just send a 30 second video and forgot to talk about some very detailed things. They won 't takes a few minutes to explain the matter thoroughly.**(P11)*  *At the beginning, there was too much and too cluttered information. I spent too much energy in going through these information. (P11)*  *I sometimes use Baidu to search for health information. Although there are some information from doctors in tertiary hospitals, but the information is very cluttered, and it 's like being pasted and copied. (P8)*  *On the Internet, some doctors say some things are actually in conflict with what other doctors say. At this time, you do not actually quite understand which situation you are applying to. (P4)*  *The videos that doctors send out, probably just don 't have much about......, they 're just rather sporadic. They also collect some other people 's questions and then send them out, so there just might not be much systematic health information. (P11)*  *For example, some video bloggers answer questions in segments. He updates one video today, and then another one tomorrow. So, I may not be able to get the information what I want in time. (P10)*  *After all, you don 't really know those people online, and then a lot of what they say, you 're not quite sure if it 's true or not, and if it 's credible or not. It 's too hard to confirm that. (P1)*  *These doctors have a lot of identities, but you simply cannot judge if you are not as a professional. Because he has some information... After all, some of these information are just for (traffic), because it is a world where traffic is king now! (P9)*  *There are some pregnant women who share their experiences about exercises on the Internet that they think are good for their bodies, or they are professional exercisers, like yogis or whatever, saying what exercises we should do in certain months of pregnancy, but this is not evidence-based. I don 't dare to do it at home. (P4)* |
| (2) Respond to barriers | *If the knowledge I have acquired from the Internet is incomplete, I would probably ask the offline doctors. Because the doctor in the hospital could give me more detailed instructions, or he could give me a list with some very detailed points to note. (P11)*  *Either that or you can register online. WeChat has online registration service! Then you can register online in the applet of WeChat, and then just ask the online doctor. (P2)*  *I will ask people who have given birth, like those classmates who have given birth to children. I would ask them if they had such reactions in the same month of pregnancy, and if these were normal. I would communicate with each other in this way. (P3)*  *I would increase search, and see if there are any pregnant women with relevant experience had any measure. Then as long as it doesn't hurt me, I would still be willing to try and then see how my body reacts. (P4)*  *When searching the web, it may be that the keywords need to be more accurate, and then it may be that multiple networks, using more than one software to query in multiple ways. (P3)*  *I usually look at a lot. If I search by myself, I wouldn't look at just one. I would look at a lot of what other people have posted and compare them. If everyone is explaining the same problem, and they all say the same solution, then you will believe that solution. (P2)*  *Another thing is that if you use Weibo, you can read the opinions of professional doctors who are recognized by the public, which I think is more authoritative, like the Weibo certification.(P7)*  *If there is something wrong or bad in what he says, someone would definitely come out and accuse him. But if he can keep updating and posting videos like this despite the scrutiny of such a large number of fans, it shows that its authenticity and reliability are relatively high. (P10)*  *I would read some of the comments underneath those about how authoritative those doctors are. (P11)* |
| 3. Desires for a higher level of online health information services |  |
| (1) Desires for online transmission media with simple design and easy-to-use search function | *I think the design of search engines and applications should be simple. Even WeChat applet or WeChat official account can be accepted. (P10)*  *I think functionality of the search must be easy-to-use. Why did I mention Little Red Booklittle just now? Because when I search for something, it could give feedback immediately.(P7)* |
| (2) Desires for diversified online transmission forms of health information | *It can be more than just words. I think it could be a little more ...... or a video communication like you. Because I think it is more intuitive. In fact, text like those on Baidu are just pieces of text information, sometimes just look at it, also can not let people cause attention,also will not let people to convince. (P1)*  *I think, have gadgets, and have text, and have video, and then have images. (P5)* |
| (3) Desires for online information platforms containing real cases and experience sharing | *There are different categories, and we can choose from them. After choosing, I can see some relevant information in them, such as some cured cases or others, so that patients can rebuild their confidence. (P10)*  *Or just like the Little Red Booklittle. There are some professional doctors in it. They have join in it. They would send some cases regularly. I think the credibility of these cases is high.(P1)*  *Because now many pregnant women like to share their experiences during pregnancy, or some records about pregnancy. In fact, you learn something from it. For example, if you are pregnant for the first time, you may be a little flustered, you don 't know anything. If you go online, you will learn a lot. (P2)* |
| (4) Desires for online information platforms with strong interactivity and personalized health information push services | *It can tell you what to pay attention to from the beginning of pregnancy and what to pay attention to later. Because if someone reminds me in the early stage of pregnancy, I would slowly control from then on to prevent GDM. (P6)*  *WeChat official account is actually quite common. I think it would be better to have a live online broadcast. Because it gives people a face-to-face feeling. Also, I can talk directly online during the live broadcast, and it would be better if there was an online Q&A session afterwards.(P4)* |
| 4. Outcomes of accessing and applying online health information |  |
| (1) Popularization of health knowledge | *I think it's quite helpful, after all, I used to have some blind spots in my knowledge, and then I downloaded Mamma Tree and learn about something, f**or example, the schedule of maternity check-ups, which is quite detailed. (P3)*  *My blood glucose is now well controlled. So in fact, it amounts to saying that, I actually gained some knowledge in the whole process. I know how I might do my blood glucose may drop a little, how I do my blood glucose may be a little higher, bring me some knowledge popularization and make me know how should Ideal with such problems in the future. (P10)* |
| (2) Emotional feedback | *It can make it more stressful. Because most people I see online might write about their relatively fortunate experiences or write about their particularly unfortunate experiences, like when she only gained 1 or 2 pounds during her entire pregnancy. That kind of can put a lot of pressure on people. I thought why can other people do this but I can 't? (P4)*  *It may also have an exaggerated element in it, because not everyone does. But it can cause some misunderstanding in others when it is simply expressed in words. I think this can have some relatively negative effects on us, and may affect our mental states, including our fears of illness, which can arise. (P10)*  *When I see what other people share on the Internet about how scary other people 's glucose is, I think that other people 's glucose is also scary, and then I feel a little less scared. (P11)* |
| (3) Increased awareness about adapting healthy lifestyles | *After a few months of control and correction, it has become very natural... For example, sweet food would not be eaten again, and then you would go to exercise after eating the staple food, which is equivalent to knowing what you can eat and what you can not eat after checking more information, and has formed a living habit. (P6)*  *Since the last time I went to check my blood glucose and it suddenly became 7.8. I have a particularly strong memory of having soup with my main meal. So, I will never forget this. Because I did neglect it before. (P4)* |
| (4) Increased husband's sense of involvement and experience | *The main thing is that it can also be shown to my husband, so that he can also feel involved. (P2)*  *There is also some information that is given to him, maybe he should do something now, maybe pay attention to give me a massage or something else, because he also has this software, he goes into his interface and he is able to see what is given to him. (P5)* |
